# Decision making preferences in the medical encounter – a factorial survey design

**DOI:** 10.1186/1472-6963-8-260

**Published:** 2008-12-17

**Authors:** Meike Müller-Engelmann, Tanja Krones, Heidi Keller, Norbert Donner-Banzhoff

**Affiliations:** 1 Department of Family Medicine, University of Marburg, Germany; 2 Bioethics/Clinical Ethics, University of Marburg, Germany

## Abstract

**Background:**

Up to now it has not been systematically investigated in which kind of clinical situations a consultation style based on shared decision making (SDM) is preferred by patients and physicians. We suggest the factorial survey design to address this problem.

This method, which so far has hardly been used in health service research, allows to vary relevant factors describing clinical situations as variables systematically in an experimental random design and to investigate their importance in large samples.

**Methods/Design:**

To identify situational factors for the survey we first performed a literature search which was followed by a qualitative interview study with patients, physicians and health care experts. As a result, 7 factors (e.g. "Reason for consultation" and "Number of therapeutic options") with 2 to 3 levels (e.g. "One therapeutic option" and "More than one therapeutic option") will be included in the study. For the survey the factor levels will be randomly combined to short stories describing different treatment situations.

A randomized sample of all possible short stories will be given to at least 300 subjects (100 GPs, 100 patients and 100 members of self-help groups) who will be asked to rate how the decision should be made. Main outcome measure is the preference for participation in the decision making process in the given clinical situation.

Data analysis will estimate the effects of the factors on the rating and also examine differences between groups.

**Discussion:**

The results will reveal the effects of situational variations on participation preferences. Thus, our findings will contribute to the understanding of normative values in the medical decision making process and will improve future implementation of SDM and decision aids.

## Background

### Shared decision making

Currently we are witnessing a change in the traditional physician-patient-relationship. Shared decision making (SDM) has become increasingly popular in the last decade [1]. Many patients no longer rely only on their physician's opinion about the best treatment. They have gained self-confidence and many of them want to participate in decisions or even be the only responsible decision maker [2-4].

Charles et al. [5] point out four conditions that must be met to classify the physician-patient interaction as shared decision making:

- there are at least two involved: the physician and the patient

- both are incorporated into the process of treatment decision making

- sharing information is the basic condition of SDM

- both agree with the decision made

### Research on SDM

Reviews demonstrate multiple favorable effects of SDM and related decision aids. SDM seems to increase patients' satisfaction, quality of life, treatment compliance, knowledge about the disease and the therapeutic options, perceived control of the disease and symptom control [6-9]. However, a recent review suggests that SDM might be useful only under certain circumstances, i.e. related to chronic illness and mental health problems [1].

Other studies analysed the effects of certain patient characteristics on his/her preferences either to participate or not to participate in the medical decision making process. In regard to socio-demographic variables, research mainly focused on age, educational level and gender. Most of the studies demonstrate a tendency for women [3,10], younger [11-13] and more highly educated people [4,14,15] to prefer more participation. Even so, there are studies which showed opposing results for gender [14] and a lack of association with educational level [16]. Increasingly, health professionals agree that all patients should be offered SDM irrespective of age, gender or formal education.

### When should SDM be used?

Whereas there have been many studies on patients' characteristics and participation preferences, the role of the treatment situation has hardly been investigated. There might be a difference whether there are severe side effects to a treatment or not, whether there are various options or only one, whether the condition to be treated is life threatening or not, on the degree to what extend SDM is regarded as appropriate or not. Up to now, most studies dealing with situational factors related to SDM preferences only focused on one dimension of the treatment situation. Furthermore, there are only a few studies addressing the normative question when SDM should be applied. Mc Kinstrey [16] confronted patients with video vignettes of consultations about five different themes (serious acute, minor acute, chronic, mental health and lifestyle), using either a shared decision or a directive consultation style. In this study, patients were more likely to prefer SDM in situations dealing with psychological problems or lifestyle. Similarly, Deber et al. [17] showed, in another vignette study, that in situations involving a potential life threatening disease there was a greater preference for giving control to the physician than in situations involving a minor disease or quality of life. If more than one treatment alternative exists, patients generally want to be involved in treatment decisions according to Guadagnoli and Ward [18].

What has been less considered is that real treatment situations are characterized by several dimensions and the complex interactions of these dimensions. Against this background, Whitney [19] developed a model addressing the normative question when SDM should be used. He characterized treatment situations by the level of importance of the medical decision for the patient and by the level of the physician's certainty about the efficacy of the therapeutic options. According to this model, decisions that are of high importance for the patient in combination with a low physician's certainty should be made by the patient, whereas the physician should make decisions of high treatment certainty with low importance for the patient. The remaining decisions should be shared.

However, this model does not include dimensions named before like the number of therapeutic options and side effects of therapeutic options. Up to know it is still not clear, which dimensions of treatment situations are important in answering the question when SDM is preferred. As a first step we suggest a factorial survey of patients and physicians.

### Analysing values in complex decisions: the role of the factorial survey

The method of the factorial survey was developed by Rossi and Anderson [20] in the eighties to study opinions, attitudes and decisions related to complex situations. It combines the strength of a classical experiment with the advantage of a sample survey design. Like an experimental design, it allows to manipulate several variables. At the same time a large number of subjects can participate without undue expense [21].

In a factorial survey, realistic vignettes, i.e. short narratives of situations or actors, are presented to the subjects. It differs from other vignette surveys in that the characteristics (factors) are varied in a systematic way [22]. Subjects rate the vignettes on a scale depending on the research question, e.g. indicating what kind of decision one should make in that specific situation. Each respondent receives a set of stories with varying combination of characteristics.

The aim of the statistical analysis is to explore the importance of each factor and interactions between different factors. Furthermore, differences in rating between respondents or groups of respondents can be analysed [22] including demographic variables [23].

Developed in the social sciences, factorial surveys have up to now been applied in many different fields proving their versatility and utility as a research method. A wide range of topics has been covered, e.g. consumer preferences [24], how people define alcohol abuse [25] and how couples decide to move [26].

Lauder [21] claims that factorial surveys help to understand the factors that underlie clinical judgments of health care professionals. Yet, there are only a few published examples in health services research. Currently, factorial surveys were occasionally undertaken in nursing science [21,27-29]. Furthermore, the method was used to investigate the reasons that make a medical error unacceptable [30], the factors influencing the reaction to medical errors [31] and how physicians decide to prescribe benzodiazepines for nervousness and insomnia [32].

### Hypothesis

We consider the factorial survey to be a feasible method to analyse the variables underlying the decision making process in the medical encounter. It will help to reveal the importance of different situational factors, determining when patients and physicians prefer SDM. Furthermore, it might serve as a tool to analyse differences between patients and physicians in this domain.

## Methods/design

### Choosing factors in a systematic way

While factors for factorial surveys are often chosen somewhat haphazardly based on literature and individual researchers' perceptions, we undertook a qualitative study to identify systematically the input for our main survey [23].

We developed an interview guideline based on published literature on situational factors related to SDM. 12 physicians, 15 patients and 14 experts in the health care system were interviewed. Among the latter there was a representative of the German Federal Ministry of Education and Research, the national Commissioner for Patients' Affairs, a speaker of The National Association of Statutory Health Insurance Physicians and a member of The Agency for Quality in Medicine.

In this qualitative study we covered three key areas: characteristics of the disease, characteristics of therapeutic options and characteristics of the patient. For each topic participants were asked to mention factors influencing the decision making process with regard to the person who should make the decision. In a second step, interviewees were asked about their opinion on the importance of the factors mentioned in the literature. The interviews were recorded, transcribed and quantitatively and qualitatively analysed using an inductive coding system.

In total we found 32 factors, 9 related to the disease i.e. severity, 8 related to the therapeutic options i.e. whether there is only one or more than one therapeutic option and 15 related to the patient i.e. the wish of the patient to participate.

To reduce the number of factors, we combined factors with similar contents and excluded those that were mentioned by only a few people. Furthermore, we excluded most patient characteristics, because the focus of our work is based on situational factors. As a result we finally achieved a 7-factor solution for the survey (see table 1).

**Table 1:** Factors and levels included in the study

	Factors	Levels
1	Reason for the consultation	• Prevention • Mild disease • Severe disease
2	Time of negative consequences	• Immediate negative • Negative in the future
3	Time until treatment should be started	• Treatment should be started immediately • For the beginning of treatment there is no time pressure
4	Number of therapeutic options	• One therapeutic option • More than one therapeutic option
5	Adverse effects of the treatment	• Treatment(s) is (are) well tolerated • Treatment(s) could have severe adverse effects • Treatments are partly well tolerated, may partly have severe adverse effects
6	Scientific evidence for the effectiveness of the treatment	• Good evidence for effect • No evidence for Effect • Good evidence for one/some treatment option/s, no evidence for other treatment option/s
7	Wish of the patient to participate	• The patient wishes to participate • The patient does not wish to participate

To construct vignettes, for each factor specific levels have to be defined. Few levels are to be preferred in order to keep the number of possible vignettes small [33]. Therefore we did not include more than 3 levels for each factor. In factors like "time until treatment should be started" we introduced only two levels, i.e. "immediate treatment necessary" vs. "no time pressure". Table 1 gives an overview of all factors and their levels finally included in our study.

### Creating vignettes

The principle of a factorial survey is to combine all levels of one factor with all levels of the other factors. Therefore, for writing a vignette, one level of a factor is randomly combined with one level of all other factors, resulting in a description of a specific situation.

Given 7 factors with 2 to 3 levels in our study, we get 432 possible vignettes by 3 × 2 × 2 × 2 × 3 × 3 × 2. Before constructing the vignettes, unrealistic combinations have to be eliminated [33]. Thus, we had to exclude the combination of "prevention" and "direct negative consequences" as there are no prevention procedures with direct negative effects in case of omission. In addition, we had to exclude vignettes containing the combination of "one therapeutic option" with either "treatments are partly well tolerated, may partly have severe adverse effects"or "good evidence for one/some treatment option/s, no evidence for other treatment option/s". Table 2 shows a possible vignette of our study.

**Table 2:** Example of a survey vignette

A patient consults his/her physician because of a mild disease that doesn't affect him right now but could lead to negative consequences in the future. There is no time pressure for beginning the treatment. There are various therapeutic options. Treatments are partly well tolerated and may partly have severe adverse effects. There is good evidence for all therapies. The patient in general wishes to participate in medical decisions.

By considering vignettes in such an abstract manner, we wondered whether all patients could imagine such situations without further details. Therefore we considered constructing vignettes with specific diseases and therapeutic options. To avoid confusion with too much detail we decided to present a semi-concrete solution as shown in table 3.

**Table 3:** Example of a semi-concrete vignette

A patient consults his doctor because of mild elevated blood pressure that doesn't affect him right now but could lead to negative consequences in the future. There is no time pressure for beginning the treatment. The available therapeutic options are: a pill that could have adverse effects or a change of lifestyle that is well tolerated. There is good evidence for all therapeutic options. The patient in general wishes to participate in medical decisions.

To find out whether the abstract (see table 2) or the semi-concrete version (see table 3) will be better accepted, better understood and easier to rate for physicians as well as for patients, we will conduct a cognitive pretest based on think aloud techniques and cognitive probes [34,35].

To rate preferences for participation in treatment decisions, we will use a 5-point scale presented by Sutherland et al. [36]. We adapted the wording so that it could be used by the patients and physicians (see table 4).

**Table 4:** The 5-point scale for rating preference for participation in treatment decision making (adapted form Sutherland et al.)

Please check the statement that best describes what you belief would be ideal:
• The doctor should make the decision using all that is known about the treatment
• The doctor should make the decision but strongly consider the patients opinion
• The doctor and the patient should make the decision together on an equal basis
• The patient should make the decision but strongly consider the doctor's opinion
• The patient should make the decision using all he knows or learns about the treatment

### Statistical considerations

There is no consensus on how many factors can be included in a factorial survey [33]. Beck and Opp [33] conclude that subjects don't have any difficulties with vignettes up to 6 dimensions, whereas Taylor [23] points out that more than twenty are possible. Nevertheless, most of the studies on factorial surveys have not used more than 8 factors [29,30,33]. Considering the capacity of the human's working memory, having difficulties to capture more than 6 to 9 information units at the same time [37], it makes sense to limit the number of factors included in the vignettes. Therefore, in our main study we will include only the 7 factors mentioned above.

Each participant will receive 11 vignettes for rating. Generally speaking, the number of vignettes for each participant depends on the time available, on the difficulty and complexity of the vignettes and on statistical considerations [33]. Previous studies used various numbers of vignettes given to each subject, ranging from only 3 [30] up to 40 [38] and even more in older investigations [39]. Beck and Opp [33] suggest that when there is no antecedent survey of a specific issue and therefore no information on how many vignettes could be tolerated, a number of 10 to 20 vignettes is reasonable. Furthermore, the number of vignettes should be higher than the number of factors and 10 vignettes seems to be the statistically best solution (C. Sauer, University of Duisburg-Essen, personal communication). Under these considerations and due to the complexity of our seven-factor survey, 11 vignettes per participant seem a reasonable choice to us. However, the ideal number of vignettes given to each subject is an additional aspect that will be tested in the pretest.

The vignettes that will be included in the study will be randomly chosen from all possible vignettes. For vignettes selection, two statistical methods are mentioned in the literature [40]. "Random designs" randomly select the desired number of vignettes from all possible vignettes. The disadvantage of this method is that main and interaction effects are confounded in the statistical analysis [22]. In "quota design" a planned selection of the vignettes is suggested to reduce this source of error. Unfortunately, quota designs in complex survey studies including many factors and different numbers of factor levels are computationally demanding and reliable statistical solutions are not available. Furthermore, quota designs are not applicable, if factor combinations have to be excluded from the study for logical reasons like in our design [40]. We therefore chose the random selection method.

Supported by a computer program 40 sets of vignettes will be randomly drawn with replacement from the pool of possible vignettes. The number of 40 results form a calculation based on the total number of vignettes, the expected sample size and the number of vignettes that will be given to each participant [33]. All 40 sets will be available for all three study groups (see "Participants"). Distribution will be at random within groups.

### Participants

To our knowledge there are no well established power analysis methods for hierarchical models in factorial survey study designs. Thus, we decided to use conservative ANOVA for fixed effects, special, main effects and interactions [41]. The sample size was calculated assuming an effect size of 0.25. Defining the single vignette as the unit of analysis, we estimated that a minimum number of 438 observations would be required to detect an effect of 0.25 with a power of 1-β = 0.95. Since one subject has to rate 11 vignettes, 40 subjects would be necessary. As we don't have estimations for intracluster correlation coefficient (ICC) and in order to compensate for drop outs and missing data, we aim at a sample size of 100 persons.

This results in a total number of 300 participants who will be included in the study: 100 general practitioners (GPs), 100 patients and 100 members of self-help groups.

We decided to include GPs because in Germany the general practitioner is the first person in contact with the patient. He is the one who guides the patient through the health care system. Usually, medical treatment is coordinated in a GP's surgery. As a consequence of the special relationship between GP and patient, most important decisions in the medical encounter are made in this context. For the study we will recruit members of our regional GP network as well as attendees of national conferences.

We plan to distinguish between the "usual" patient seen in general practice and members of self-help groups because we expect the latter to be more active in the decision making process. Patients will be recruited in surgeries. Members of self-help groups will also be contacted resorting to self-help group nets.

Patients are included, if they are 18 years and older, able to read German and able to give informed consent. We try to cover different educational levels and age groups, balanced for gender. Patients requiring emergency care are excluded.

### Material given to the participants

Before receiving the vignettes, all participants will be given written information about the study and will have to sign an informed consent form, approved by the local research ethics committee, University of Marburg. Participants will then fill in the survey questionnaire. After asking for socio-demographic data and giving instructions 11 vignettes will be presented with the response format described above. If the participants have any questions a member of the research team will be present.

### Data analysis

For our analysis we will use mixed models to estimate the effects of factors and interactions. Factors will be coded as dummy variables. In addition, we will examine differences between the three groups (physicians, practice patients and members of self help groups). Furthermore, we will investigate the influence of participants' demographic characteristics.

Linear models based on Ordinary Least Squares (OLS) techniques, e.g. analysis of variance or multiple regression, cannot be used for analysis since they would assume independence of all judgements [21]. However, responses in factorial surveys are clustered in subjects [42]. Therefore, hierarchical models based on maximum likelihood estimators seem to be more appropriate for this design. Hox et al. [42] demonstrated that choice between OLS-estimation and hierarchical/mixed models is critical since there are large calculational differences between the two solutions.

## Discussion

The findings of this factorial survey study will reflect normative values related to medical decision making. They will reveal the effect of situational variations on consultation style preferences.

The results might help physicians to adapt their consultation style not only to individual patients but also to individual treatment situations. Future implementation of SDM and decision aids might be more successful than in the past when communication patterns are tailored to specific situations.

Furthermore, we hope to encourage researchers of the effects of SDM to incorporate situational factors into their study designs.

We believe that the factorial survey will become a much more frequently used study design. A pure top-down approach to meso- and macro-level-decisions in health care is becoming less acceptable. In most instances many stakeholders are involved ranging from patients to providers, payers and governments, to commercial manufacturers of drugs and devices. Factorial survey designs can make an invaluable contribution, since opinions, interests and values of multiple stakeholders are systematically assessed to inform policy decisions in health care.

## Competing interests

The authors declare that they have no competing interests.

## Authors' contributions

All authors have contributed to the development of the study design. TK and NDB sought funding. MME is responsible for the management of the trial assisted by HK. MME has drafted the manuscript with input from the other authors who have read and approved the final version.

## Pre-publication history

The pre-publication history for this paper can be accessed here:


